# Comprehensive Characterization of Metabolites in Multiplier Onion Bulbs and Identification of Regulatory Genes for Nutritional Improvement

**DOI:** 10.3390/foods14193290

**Published:** 2025-09-23

**Authors:** Huixia Jia, Jiangping Song, Tingting Zhang, Yumin Tan, Mengzhen Wang, Jiyan Zang, Xiaohui Zhang, Wenlong Yang, Yanhui Pang, Yanfei Yang, Haiping Wang

**Affiliations:** State Key Laboratory of Vegetable Biobreeding, Institute of Vegetables and Flowers, Chinese Academy of Agricultural Sciences, Beijing 100081, China; jiahuixia@caas.cn (H.J.); songjiangping@caas.cn (J.S.); zhangtingting@caas.cn (T.Z.); Tanyumin1218@163.com (Y.T.); 17332327872@163.com (M.W.); zangjiyan2001@163.com (J.Z.); zhangxiaohui01@caas.cn (X.Z.); yangwenlong@caas.cn (W.Y.); 18324697681@163.com (Y.P.); 13759225665@163.com (Y.Y.)

**Keywords:** multiplier onion, metabolome, transcriptome, nutritional composition, regulatory genes

## Abstract

Multiplier onion (*Allium cepa* var. *aggregatum*) is an economically important Allium crop that serves dual purposes as both a culinary ingredient and medicinal resource. Despite its widespread utilization, systematic characterization of its nutrient metabolism components remains limited, which has constrained the development of high-value cultivars with optimized nutritional profiles. In this study, we conducted a comprehensive metabolomic profiling of bulbs from five genetically distinct accessions using a widely targeted metabolomic method based on ultra-performance liquid chromatography–tandem mass spectrometry (UPLC-MS/MS). The analysis identified 659 metabolites, including lipids, flavonoids, phenolic acids, amino acids, saccharides and alcohols, organic acids, alkaloids, nucleotides and derivatives, vitamins, etc. Notably, the bulbs exhibited a high abundance of flavonoids (e.g., quercetin, kaempferol, naringenin, isorhamnetin) and eight essential amino acids (valine, threonine, leucine, isoleucine, lysine, methionine, histidine, and tryptophan). Comparative analysis revealed that 366 differentially accumulated metabolites were identified among these 5 accessions, many of which were significantly enriched in pathways related to flavonoid biosynthesis, as well as amino acid biosynthesis and metabolism. Transcriptome analysis indicated that differentially expressed genes (DEGs) across the five accessions were significantly enriched flavonoid biosynthesis, and various amino acid biosynthesis and metabolism processes, such as “tyrosine metabolism”, “phenylalanine, tyrosine, and tryptophan biosynthesis”, “cysteine and methionine metabolism” and “arginine and proline metabolism”, being consistent with the substantial variations observed in flavonoids, amino acids, and their derivatives in the metabolome analysis. Correlation and network analysis identified several crucial candidate regulatory genes for the biosynthesis and metabolism of lipids, flavonoids, phenolic acids, and amino acids. These findings provide a comprehensive metabolic atlas of multiplier onion, reveal key genetic regulators of nutritional quality, and establish a scientific foundation for nutraceutical development and cultivar improvement strategies.

## 1. Introduction

Onions, which belong to the *Allium* genus within the Liliaceae family, are important commercial horticultural crops widely utilized for culinary, condiment and medicinal purposes. Cultivated onions can be categorized into two main types: the common onion (*Allium cepa*) and the multiplier onion (*Allium cepa* var. *aggregatum*). The common onion typically yields a single large bulb per plant and reproduces sexually through seeds. In contrast, the multiplier onion, also referred to as shallot, potato onion, small onion or aggregatum onion, produces between three and twenty small bulbs per plant and primarily propagates asexually through bulbs [1]. Compared to the common onion, the multiplier onion has a shorter growth cycle of approximately three months, a longer storage life, and greater resistance to pests, diseases, and environmental stresses [2]. Its primary edible organ is the bulb, which is available in various forms, including fresh, frozen, canned, pickled and chopped. Currently, the multiplier onion is cultivated in Europe, South America, and Asia [3].

Onions are an excellent dietary source of nutrients and phytochemicals [4,5]. In addition to minerals, vitamins, and fiber, onions are particularly valued for their rich polyphenolic composition, such as phenolic acids and flavonoids [6]. These compounds demonstrate potent antioxidant capacity and anti-inflammatory activity, contributing to their documented protective effects against chronic diseases including cardiovascular disorders and certain cancers [7,8]. To date, extensive research efforts have focused on elucidating the metabolic composition of common onion, particularly with regard to polysaccharides [9], sucrose [10], and anthocyanin [11]. Comprehensive metabolite profiling has been extensively studied in common onion cultivars utilizing various analytical methods. For example, liquid chromatography coupled with electrospray ionization quadrupole time-of-flight mass spectrometry (LC/ESI-QTOF-MS) has facilitated the detection of 123 metabolites in onion bulbs, including fructooligosaccharides, proteinogenic amino acids, peptides, S-substituted cysteine conjugates, flavonoids, and saponins [12]. A combined gas chromatography–mass spectrometry (GC-MS) and ultra-performance liquid chromatography-mass spectrometry (UPLC-MS) metabolomic approach applied to 4 Egyptian onion cultivars identified 117 metabolites, encompassing flavonoids, nitrogenous compounds, fatty acids, and silylated primary metabolites, highlighting their chemical diversity and significant antimicrobial activity against foodborne pathogens [13]. Furthermore, LC-ESI-QTOF-MS was used in 9 onion cultivars for the identification and relative quantification of 106 metabolites, demonstrating that genetic factors predominantly influence saccharide and flavonoid content, while both genetic and environmental factors significantly affect the levels of cysteine sulfoxides, amino acids, and peptides [14]. These studies offer valuable insights into the culinary potential, nutritional value, health benefit, and phytochemical diversity of common onion accessions.

Preliminary studies indicate that multiplier onion contains abundant flavonoids and polyphenols [3]. The phenolic content in multiplier onion is observed to be over 50% higher than that in bulb onions [15]. Furthermore, the volatiles including aldehydes, alcohols, pyrazines and sulfur-containing compounds have been identified in fried multiplier onion [16]. However, the panoramic metabolic profile and nutritional composition of multiplier onion have not yet been fully characterized, and the regulatory mechanisms of key metabolites remind unexplored. Metabolomics is the comprehensive analysis and measurement of all metabolites in a given organism or biological sample [17]. These metabolites determine the nutritional and consumption value of the crops [18]. With the advancement of metabolite profiling technologies, widely targeted metabolomics has emerged as a powerful approach that combines the broad screening capacity of untargeted methods with the accurate quantification of targeted analysis. This method offers high throughput and high sensitivity, enabling effective determination of metabolites, and has been successfully applied to plant metabolite analysis across multiple species [17,18,19]. Thus, widely targeted metabolomics provides a powerful tool to decode the comprehensive metabolic profiling of multiplier onion and to identify molecular targets for quality improvement.

In the present investigation, we employed an integrated multi-omics approach to systematically characterize the metabolic components and gene expression profiles among five genetically distinct accessions of multiplier onion. Utilizing a widely targeted metabolomic method based on ultra-performance liquid chromatography–tandem mass spectrometry (UPLC-MS/MS), we conducted comprehensive metabolic profiling to identify accession-specific differential metabolites. Furthermore, Illumina RNA-seq data were utilized to compare gene expression differences among the various accessions, thereby elucidating gene–metabolite relationships. This study aims to establish the first metabolic atlas of multiplier onion and uncover key regulatory networks underlying nutrient accumulation. These findings provide valuable insights into the components of nutrient metabolism and strategies for the improvement of multiplier onion varieties.

## 2. Materials and Methods

### 2.1. Plant Materials

**Figure 1:**
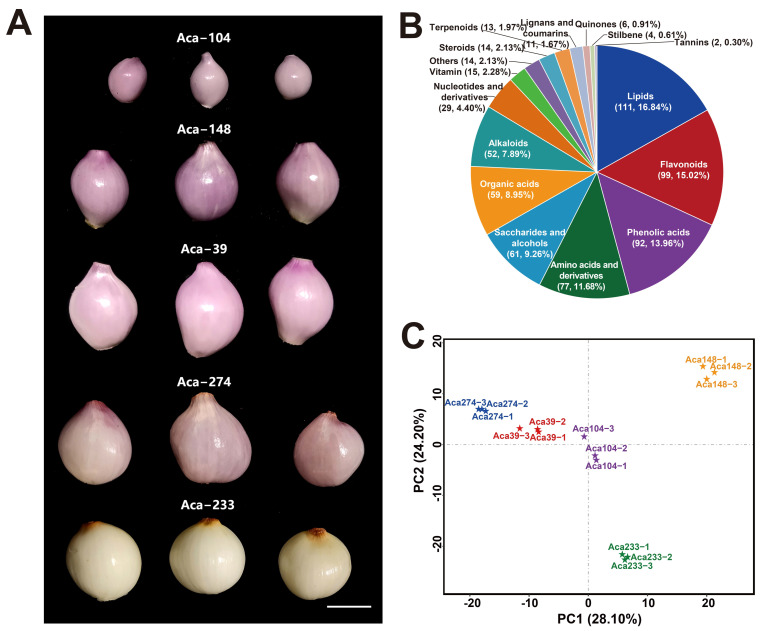
**Metabolic profiling analysis of five accessions of multiplier onion.** (**A**) Five accessions (Aca−39, Aca−104, Aca−148, Aca−233, and Aca−274) were selected for UPLC-MS/MS metabolomics analysis. (**B**) Sixteen groups of metabolites, including lipids, flavonoids, phenolic acids, amino acids and derivatives, saccharides and alcohols, organic acids, alkaloids, nucleotides and derivatives, vitamin, steroids, terpenoids, lignans and coumarins, quinones, stilbene, tannins, and other categories. (**C**) Principal component analysis of metabolites in the five accessions of multiplier onion.

Five genetically distinct accessions of multiplier onion (Aca−39, Aca−104, Aca−148, Aca−233, and Aca−274) (Figure 1A) were subjected to UPLC-MS/MS detection and Illumina RNA sequencing. These materials were cultivated in the experimental fields of the Vegetable Research Center at the International Agricultural High-tech Industrial Park of the Chinese Academy of Agricultural Sciences. For each accession, a uniform size of 30 bulbs was sown in the soil, with row spacing set at 10 cm and column spacing at 20 cm. Field cultivation was carried out in accordance with standard agricultural practices.

### 2.2. Metabolite Extraction

Bulbs of five accessions were freeze-dried with the aid of a vacuum freeze-dryer (Scientz-100F, Scientz Corporation, Ningbo, China). The freeze-dried samples were then processed by crushing them in a mixer mill equipped with a zirconia bead for 1.5 min at a frequency of 30 Hz. An amount of 100 mg of the lyophilized powder was dissolved in 1.2 mL of a 70% methanol solution. The sample was vortexed every 30 min for 30 s each time, repeated 6 times in total. It was then stored overnight at 4 °C and subsequently centrifuged at 12,000 rpm for a duration of 10 min. The resultant extracts were subsequently filtered through a filter (Millipore Corporation, Bedford, MA, USA) with a pore size of 0.22 μm into prepare for widely targeted metabolomics analysis based on UPLC-MS/MS. Each accession was analyzed with three independent biological replicates, resulting in a total of 15 samples subjected to widely targeted metabolomics analysis.

### 2.3. UPLC-MS/MS Detection

The data acquisition instrument system primarily consists of an ultra-performance liquid chromatography (UPLC) system (SHIMADZU Nexera X2, Shimadzu Corporation, Kyoto, Japan) and a tandem mass spectrometry (MS/MS) system (4500 QTRAPApplied Biosystems, Foster City, CA, USA). Liquid chromatography was performed using an Agilent SB-C18 column (2.1 mm × 100 mm, 1.8 µm particle size, Agilent Technologies, Santa Clara, CA, USA). The mobile phase consisted of ultrapure water with 0.1% formic acid (Phase A) and acetonitrile with 0.1% formic acid (Phase B). The elution gradient was set as follows: at 0.00 min, the proportion of Phase B was 5%; over 9.00 min, the proportion of Phase B increased linearly to 95%, remaining at 95% for 1 min; from 10.00 to 11.10 min, the proportion of Phase B decreased to 5%, equilibrating at this level until 14 min. The flow rate was set at 0.35 mL per minute, with the column oven temperature maintained at 40 °C, and the injection volume set to 4 μL. Both linear ion trap (LIT) and triple quadrupole (QQQ) scans were obtained using a triple quadrupole-linear ion trap mass spectrometer (Q TRAP, Applied Biosystems Sciex, Framingham, MA, USA), the AB4500 Q TRAP UPLC/MS/MS System(Applied Biosystems Sciex, Framingham, MA, USA), equipped with an ESI Turbo Ion-Spray interface (Applied Biosystems Sciex, Framingham, MA, USA), operated in both positive and negative ion modes and controlled with Analyst 1.6.3 software (Applied Biosystems Sciex, Framingham, MA, USA). The ESI source parameters were set as follows: ion source set to turbo spray; source temperature at 550 °C; ion spray voltage (IS) at 5500 V (positive ion mode)/−4500 V (negative ion mode); ion source gas I (GSI), gas II (GSII), and curtain gas (CUR) were set at 50, 60, and 25.0 psi, respectively; the collision gas (nitrogen) was set to medium; and collision-induced ionization parameter was set to high. Instrument tuning and mass calibration were conducted using polypropylene glycol solutions at concentrations of 10 and 100 μmol/L in QQQ and LIT modes, respectively. QQQ scans were acquired through MRM experiments, with the collision gas (nitrogen) set to medium. Declustering potential (DP) and collision energy (CE) for individual MRM transitions were further optimized. Based on the metabolites eluted within each period, a specific set of MRM ion pairs was monitored for each period.

### 2.4. Differentially Accumulated Metabolites Analysis and KEGG Enrichment

Metabolite identification was performed based on an in-house database, MWDB (MetWare Database), by matching secondary mass spectrometry data. During the analysis, isotopic signals were excluded, as well as duplicate signals originating from K^+^, Na^+^, and NH_4_^+^ adducts, and fragment ions derived from higher-molecular-weight compounds. Metabolite quantification was carried out using the multiple reaction monitoring (MRM) mode on a triple quadrupole mass spectrometer. Mass spectrum data were analyzed using the Analyst 1.6.3 software. After acquiring the metabolic mass spectrum data, integration of peak areas was carried out for all peaks related to the substances, with modifications applied to ensure consistency across samples for the mass spectrum peaks of the same metabolites. Both qualitative and quantitative assessments of the metabolites were achieved through mass spectrometry. The MetaboAnalystR (v4.0) package was used to derive the VIP values from the results of the OPLS-DA, which additionally included score and permutation plots [20]. Before conducting OPLS-DA, the dataset was log_2_-transformed and mean-centered. To avoid overfitting, a permutation test consisting of 200 permutations was executed. The screening criteria of differentially accumulated metabolites (DAMs) are as follows: metabolites with fold change ≥ 2 or fold change ≤ 0.5 are selected, along with those exhibiting a VIP value ≥ 1. Unsupervised PCA using the prcomp function in R (v4.2.0) package was conducted on relative quantitative data of all identified metabolites from the 15 samples. The hierarchical clustering of DAMs was represented through heatmaps with corresponding dendrograms. MetaboAnalyst (v5.0), along with the KEGG database, was employed to investigate metabolic pathways related to the differential metabolites, and the significance of these pathways was evaluated using a hypergeometric test [21].

### 2.5. RNA Extraction and Illumina RNA Sequencing

RNA sequencing using Illumina technology was performed on the bulbs of five different accessions (Aca−39, Acav104, Aca−148, Aca−233, and Aca−274) in order to analyze their gene expression profiles. RNA was extracted utilizing the Tiangen RNA preparation kit (Tiangen Biotech (Beijing) Co., Ltd, Beijing, China). The cDNA libraries were constructed with the NEBNext Ultra RNA Library Prep Kit (New England Biolabs Inc., Ipswich, MA, USA), along with NEBNext Multiplex Oligos designed for Illumina (Illumina, Inc., San Diego, CA, USA). The enriched mRNA was fragmented into RNA segments of approximately 400 bp, which were subsequently used for cDNA synthesis. End-repair, dA-tailing, and adapter ligation were performed on the double-stranded cDNA. AMPure XP beads were employed to isolate suitable fragments, which were then enriched through PCR amplification. Ultimately, the product from the cDNA library was sequenced on the Illumina HiSeq2500 platform (Illumina, Inc., San Diego, CA, USA), achieving a read length of 150 bp. Three biological replicates were carried out independently per accession, yielding a total of 15 samples for RNA sequencing analysis.

### 2.6. Gene Differential Expression Analysis and Gene Ontology Enrichment

A Perl script was employed to eliminate low-quality reads, which consisted of those that contained solely adapters, those with over 5% unknown nucleotides, or Q20 scores falling below 20%. The refined reads were aligned with full-length transcripts identified through PacBio SMRT sequencing, employing Tophat2 software (v2.1.1) [22]. The alignment results, in BAM/SAM format, were further scrutinized to eliminate any potential duplicate molecules. Cufflinks software (v2.2.1) was then used to measure gene expression levels in terms of FPKM values [23]. The DESeq2 package (v3.16) [24] was applied to assess gene expression variations across five chosen accessions. Genes showing significant differential expression (DEGs) were defined as having an absolute log_2_ fold change (|Log_2_FoldChange|) of ≥1 and a false discovery rate (FDR) value of <0.05. For the analysis of metabolic pathways, the Kyoto Encyclopedia of Genes and Genomes (KEGG) program was utilized, and Fischer’s exact test was performed to assess the significance of the KEGG pathways.

### 2.7. Integration Analysis of Metabolomics and Transcriptomics

An analysis integrating metabolomics and transcriptomics data was performed to identify genes related to metabolites. To evaluate the relationship between DAMs and DEGs, Pearson’s correlation coefficients (PCCs) were employed. The evaluation of correlation utilized the “cor” and “corPvalueStudent” functions from the R (v4.2.0) package. A correlation was considered significant if the absolute value of the correlation coefficient surpassed 0.90 and the *p*-value was below 0.05. The correlation network was visualized using Cytoscape (v3.7.1) [25].

## 3. Results

### 3.1. Metabolic Profiling Analysis

Utilizing the widely targeted metabolomic method, 659 metabolites were identified in the 5 accessions of multiplier onion (Figure 1A). The Total Ion Chromatogram (TIC) of all 15 samples and the MS/MS spectra of several metabolites were shown in Figure S1 and Figure S2, respectively. These metabolites were categorized into 16 groups (Figure 1B, Table S1). The most prevalent metabolites included lipids (111, 16.84%), followed by flavonoids (99, 15.02%) and phenolic acids (92, 13.96%) (Figure 1B). Notably, a significant presence of amino acids and their derivatives (77, 11.68%) was observed in the bulbs, particularly highlighting eight essential amino acids, including valine, threonine, leucine, isoleucine, lysine, methionine, histidine, and tryptophan (Figure 1B, Table S1). Additionally, 15 vitamins were detected in the bulbs, primarily comprising B vitamins such as vitamin B2, B3, B5, B7, and B9, along with vitamin E and vitamin K2 (Figure 1B, Table S1). Principal component analysis (PCA) of metabolites in the five accessions of multiplier onion revealed distinct separation of the Aca−148 and Aca−233 accessions from the other three accessions (Figure 1C).

### 3.2. Differentially Accumulated Metabolites Analysis

**Figure 2:**
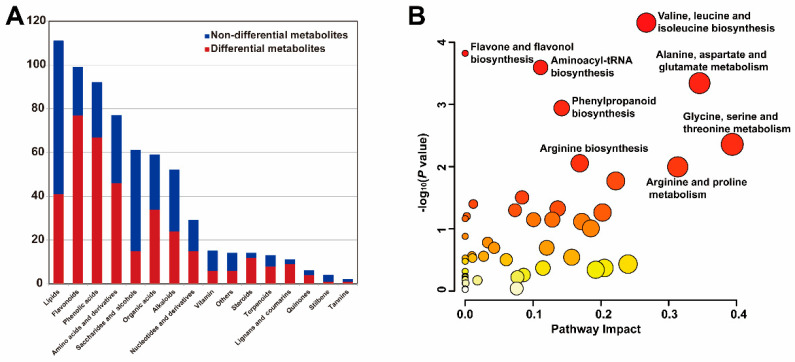
**Quantitative statistical analysis and enrichment analysis of differentially accumulated metabolites (DAMs).** (**A**) Quantitative distribution of the DAMs. (**B**) KEGG pathway analysis of the DAMs. The matched pathways were represented by circles. The color gradients of circles represent −log_10_ (*p* value) values, with darker colors indicating more significant changes in the metabolites associated with the corresponding pathway. Furthermore, the sizes of the circles correspond to the pathway impact scores.

A total of 366 differentially accumulated metabolites (DAMs) were identified across the 5 representative accessions (Table S2). Notably, more than half of the metabolites in several categories, including flavonoids, phenolic acids, amino acids and derivatives, organic acids, steroids, terpenoids, lignans and coumarins, and quinones, exhibited significant differences (Figure 2A). Among these accessions, Aca−233 demonstrated a high accumulation of most metabolites across the categories of flavonoids, phenolic acids, lipids, amino acids and derivatives, organic acids, nucleotides and derivatives, saccharides and alcohols. In contrast, Aca−148 contained a majority of metabolites, including flavonoids, phenolic acids, lignans and coumarins, while Aca−39 and Aca−274 exhibited lower metabolite content across most categories (Table S2). These findings indicate substantial variability in metabolite content among the accessions, highlighting the necessity of selecting varieties with high nutritional quality through precise detection.

To further characterize the major metabolic pathways involving these DAMs, we conducted KEGG pathway enrichment and pathway topology analysis. The DAMs were significantly enriched (*p* < 0.05) in eight pathways, five of which were related to amino acid biosynthesis and metabolism. These metabolic pathways included “valine, leucine and isoleucine biosynthesis”, “alanine, aspartate and glutamate metabolism”, “glycine, serine and threonine metabolism”, and “arginine and proline metabolism”, “arginine biosynthesis”, indicating notable variations in amino acids among the multiplier onion accessions. Additionally, the pathways for “aminoacyl-tRNA biosynthesis”, “phenylpropanoid biosynthesis”, and “flavone and flavonol biosynthesis” were also significantly enriched (Figure 2B).

### 3.3. Transcriptome Sequencing and Differential Expression Gene Analysis

**Figure 3:**
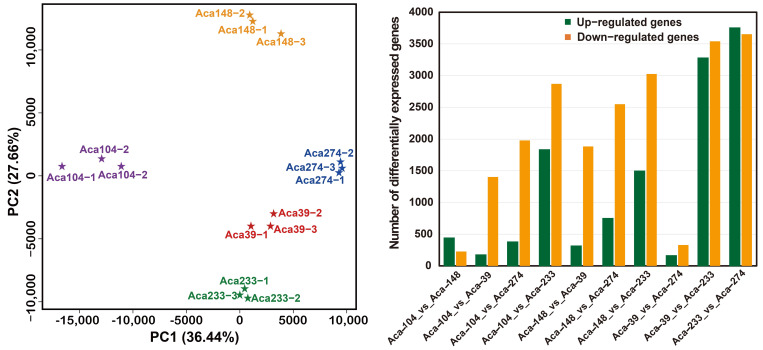
**Transcriptomic profiling of five accessions of multiplier onion.** (**A**) Principal component analysis of gene expression profiles of the five accessions. (**B**) Quantitative statistical analysis of the differential expression genes (DEGs).

To further elucidate the regulatory mechanism and metabolite-related gene expression in multiplier onion, transcriptome sequencing was conducted on the bulbs of five core garlic accessions (Aca−39, Aca−104, Aca−148, Aca−233, and Aca−274). Principal component analysis of the entire gene expression dataset revealed a tight clustering among the three biological replicates, indicating the reliability of the RNA sequencing dataset (Figure 3A). Among these five accessions, the comparable groups (Aca-104_vs_Aca-148, Aca-39_vs_Aca-274) exhibited a small number of DEGs, while the other groups (Aca−104_vs_Aca−233, Aca−148_vs_Aca−233, Aca−39_vs_Aca−233, AcaV233_vs_Aca−274) displayed a larger number of DEGs, ranging from 4704 to 7409 (Figure 3B). This observation suggested that the gene expression levels of Aca−233 differed significantly from those of the other four accessions.

**Figure 4:**
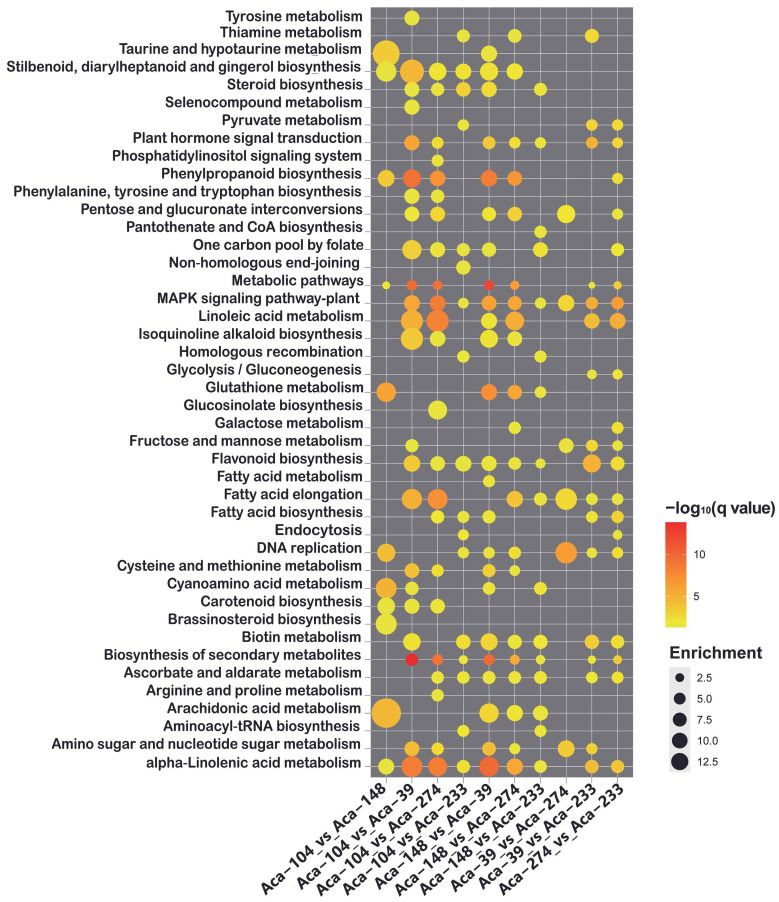
**KEGG enrichment analysis of differential expression genes (DEGs).** The pathways that exhibited significant enrichment were marked with circles. The color and size of each circle corresponded to the −log_10_ (q value) and enrichment scores, respectively. A higher −log_10_ (q value) and enrichment score signified a greater level of enrichment.

KEGG enrichment analysis indicated that the DEGs were significantly enriched in 43 metabolic pathways, including flavonoid biosynthesis and various amino acid biosynthesis and metabolism processes, such as “tyrosine metabolism”, “phenylalanine, tyrosine, and tryptophan biosynthesis”, “cysteine and methionine metabolism” and “arginine and proline metabolism” (Figure 4). This finding was consistent with the substantial variations observed in flavonoids, amino acids, and their derivatives in the metabolome analysis. Furthermore, the DEGs were enriched in pathways related to “carotenoid biosynthesis”, “brassinosteroid biosynthesis”, “plant hormone signal transduction” and “MAPK signaling pathway-plant” (Figure 4). These results indicated notable differences in multiple metabolic pathways among the multiplier onion accessions, resulting in significant variations in metabolite products across different accessions.

### 3.4. Correlation and Network Analysis of DAMs and DEGs

**Figure 5:**
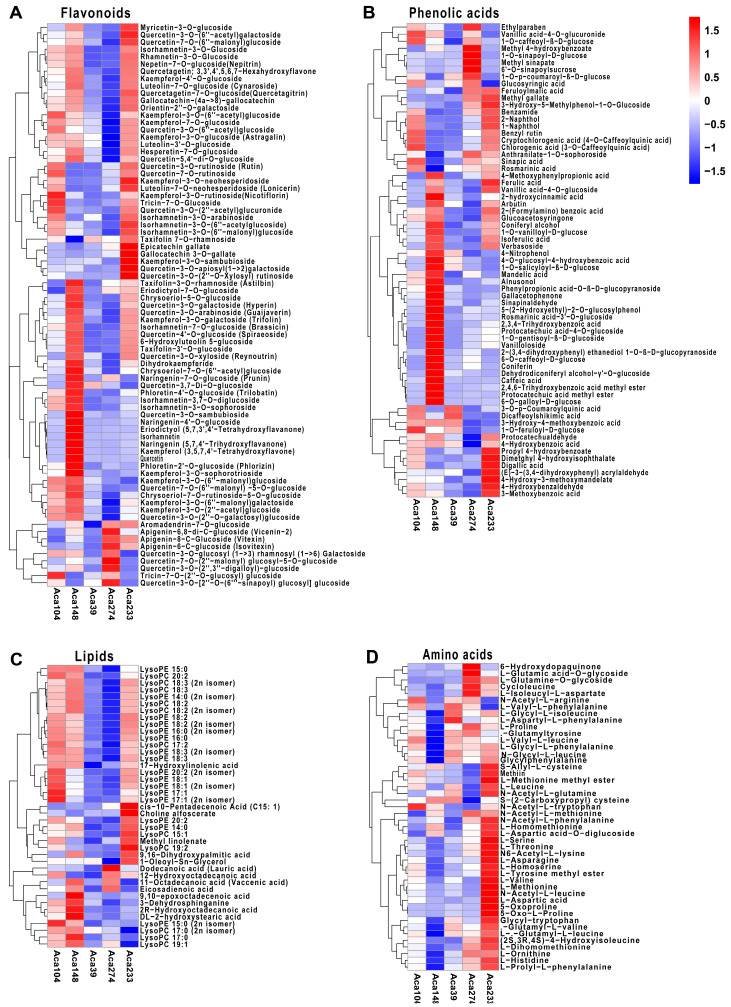
**Hierarchical clustering analysis (HCA) of the DAMs across the five accessions.** (**A**) HCA of lipids. (**B**) HCA of phenolic acids. (**C**) HCA of flavonoids. (**D**) HCA of amino acids and derivatives. The rows represent the metabolites. The colors reflect the Z-score conversion values of fold changes among the five accessions. Elevated levels of metabolites are denoted by red, whereas decreased levels are indicated by blue.

**Figure 6:**
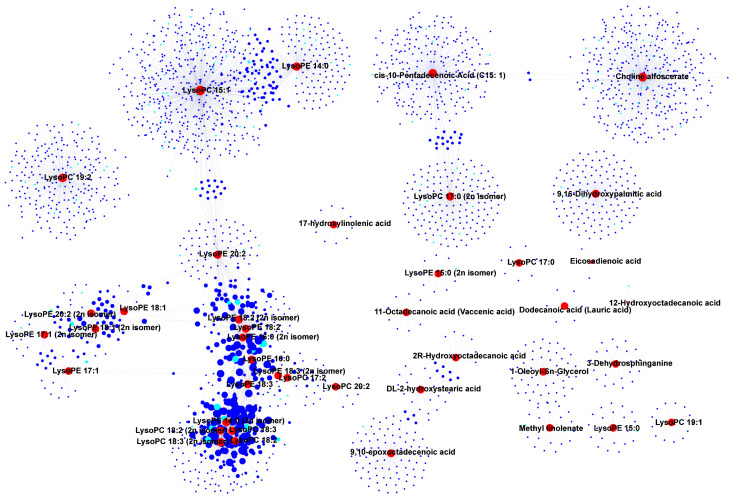
**Correlation and network analysis of differentially accumulated lipids and differentially expressed genes (DEGs).** The red circles denote the differentially accumulated lipids, while the blue circles represent the DEGs. Additionally, the sky-blue circles indicate transcription factors within the DEGs.

In the integration study of metabolomics and transcriptomics, correlation analysis was performed to elucidate the relationship between DAMs and DEGs. A correlation network was constructed to identify key genes that regulate the metabolites. Among the lipids, lysophosphatidyl ethanolamine (LysoPE 14:0, 16:0, 17:1, 18:1, 18:2, 18:3, 20:2) and lysophosphatidylcholine (LysoPC 17:2, 18:2, 18:3, 20:2), which shared multiple DEGs, were found to be highly accumulated in the Aca-104 and Aca-233 accessions (Figure 5A and Figure 6). Several of these DEGs were annotated as being involved in lipid transport and metabolism, like *acetyl-coenzyme A carboxylase carboxyl transferase subunit alpha* (*AccA*, *Aca_transcript_73117*, *Aca_transcript_92754*), and *3-hydroxyisobutyryl-CoA hydrolase 1-like* (*HIBCH*, *Aca_transcript_45116*) (Figure 6, Table S3). Additionally, some DEGs were annotated as transcription factors, such as *GATA4-like* (*Aca_transcript_38229*), *NAC68-like* (*Aca_transcript_87732*), *NAC83-like* (*Aca_transcript_58714*), *ERF38-like* (*Aca_transcript_78220*), *ERF54-like* (*Aca_transcript_14163*), and *ERF71-like* (*Aca_transcript_21000*).

**Figure 7:**
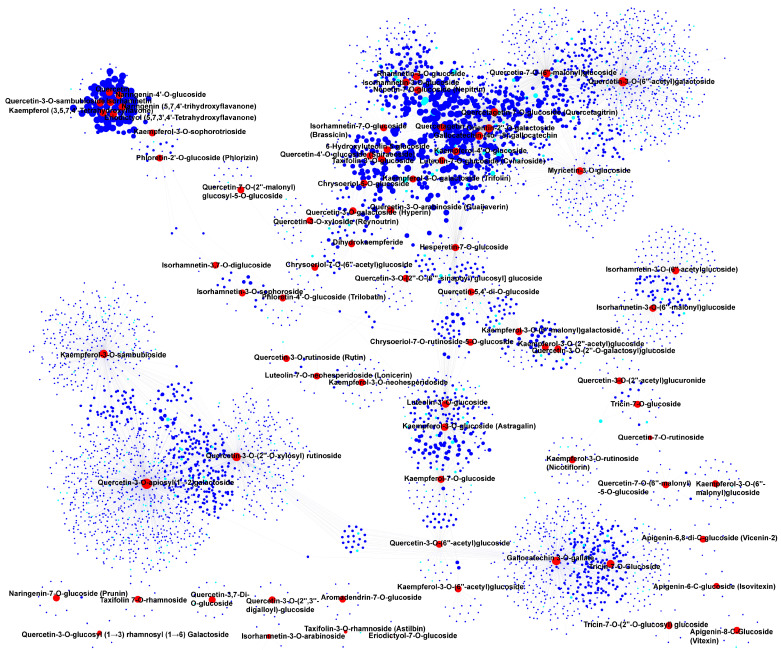
**Correlation and network analysis of differentially accumulated flavonoids and differentially expressed genes (DEGs).** The red circles denote the differentially accumulated flavonoids, while the blue circles represent the DEGs. Additionally, the sky-blue circles indicate transcription factors within the DEGs.

In flavonoids, quercetin, quercetin-3-*O*-sambubioside, naringenin, naringin-4′-*O*-glucoside, isorhamnetin, eriodictyol, and kaempferol were found to be highly accumulated in the Aca-148 accession (Figure 5B). These DAMs were associated with some co-related genes that might be involved in flavonoid biosynthesis, including *flavonoid 3′-hydroxylase* (*Aca_transcript_11093*), *glutathione S-transferase* (*Aca_transcript_21608*), and *cytochrome P450* (*Aca_transcript_71174*, *Aca_transcript_56523*) (Figure 7, Table S3). Additionally, rhamnetin-3-*O*-glucoside, isorhamnetin-3-*O*-glucoside, nepetin-7-*O*-glucoside (nepitrin), quercetin-7-*O*-(6″-malonyl) glucoside, quercetagetin-7-*O*-glucoside (quercetagitrin), quercetagetin, orientin-2″-*O*-galactoside, gallocatechin-(4α→8)-gallocatechin, kaempferol-4′-*O*-glucoside, luteolin-7-*O*-glucoside (cynaroside), myricetin-3-*O*-glucoside, and kaempferol-3-*O*-galactoside (trifolin), which shared multiple DEGs, were found to be highly accumulated in the Aca-233 accession (Figure 5B and Figure 7, Table S3). Some of these DEGs were annotated as participating in flavonoid biosynthesis and metabolism, including *flavanone 3-hydroxylase* (*Aca_transcript_81295*), *UDP-glycosyltransferase 88F3-like* (*Aca_transcript_53978*, *Aca_transcript_9276*), *flavanone 3-hydroxylase* (*Aca_transcript_17236*), *CHI-ACAD* (*Aca_transcript_22037*), and *flavonol synthase* (*Aca_transcript_17453*). Furthermore, some of these DEGs were annotated as transcription factors, such as *bZIP39-like* (*Aca_transcript_20493*), *bZIP53-like* (*Aca_transcript_18794*), *ERF12-like* (*Aca_transcript_21912*), *WRKY34* (*Aca_transcript_48555*), and *WRKY24* (*Aca_transcript_35277*).

**Figure 8:**
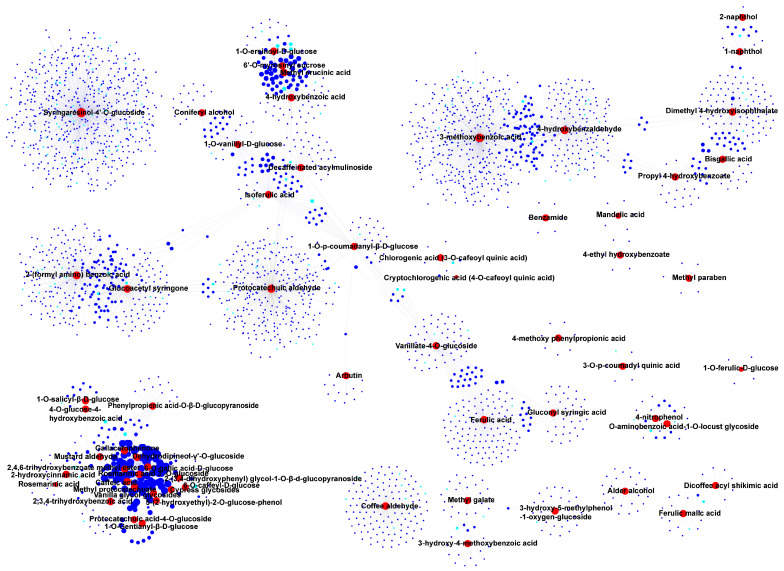
**Correlation and network analysis of accumulated phenolic acids and differentially expressed genes (DEGs).** The red circles denote the differentially accumulated phenolic acids, while the blue circles represent the DEGs. Additionally, the sky-blue circles indicate transcription factors within the DEGs.

In phenolic acids, nearly half of DAMs were found to be highly accumulated in the Aca-148 accession, like sinapinaldehyde, 6-*O*-galloyl-d-glucose, caffeic acid, coniferin, rosmarinic acid-3′-*O*-glucoside, 2,3,4-trihydroxybenzoic acid, protocatechuic acid methyl ester, protocatechuic acid-4-*O*-glucoside, and 1-*O*-gentisoyl-β-d-glucoside (Figure 5C). These DAMs were associated with several correlated genes annotated as being involved in transcription, secondary metabolite biosynthesis, transport and catabolism. Notable genes included *MYB86-like* (*Aca_transcript_96952*), *NAC 29-like* (*Aca_transcript_18633*), *cytochrome P450 94A1-like* (*Aca_transcript_47743*), *cytochrome P450 85A1-like* (*Aca_transcript_71174*), *cytochrome P450 71A1-like* (*Aca_transcript_11262*), and *cytochrome P450 81E8-like* (*Aca_transcript_56523*) (Figure 8, Table S3).

**Figure 9:**
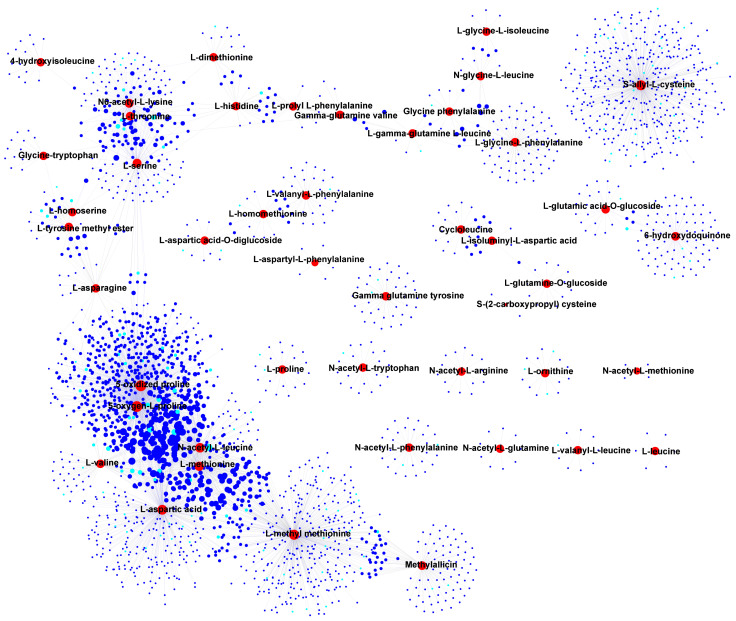
**Correlation and network analysis of differentially accumulated amino acids and derivatives and differentially expressed genes (DEGs).** The red circles denote the differentially accumulated amino acids and derivatives, while the blue circles represent the DEGs. Additionally, the sky-blue circles indicate transcription factors within the DEGs.

In amino acids and their derivatives, most DAMs were highly accumulated in the Aca-233 accession (Figure 5D). Notably, essential amino acids, including l-histidine, l-leucine, l-valine, l-threonine, and l-methionine, exhibited significant correlations with 346 DEGs, 17 of which were annotated as being involved in amino acid synthesis, transport, and metabolism (Figure 9, Table S3). These genes included *amino acid transporter 1-like* (*NAT1-like*, *Aca_transcript_57289*), *amino acid permease 3* (*AAP3*, *Aca_transcript_12428*), *amino acid permease 7* (*AAP7*, *Aca_transcript_9530*), *amino-acid permease BAT1* (*Aca_transcript_80226*), and *vacuolar amino acid transporter 1-like* (*AAT1-like*, *Aca_transcript_48521*). Three of these five genes were also co-correlated with other amino acids and derivatives. Specifically, *NAT1-like* was co-correlated with N6-acetyl-l-lysine, *AAP7* with l-Aspartic acid, and *AAT1-like* with l-prolyl-l-phenylalanine. Additionally, some genes were found to be co-correlated with multiple amino acids and derivatives. For instance, *isoaspartyl peptidase/*l*-asparaginase 1-like* (*Aca_transcript_29368*) was co-correlated with l-Homoserine, l-Asparagine, and l-Tyrosine methyl ester.

## 4. Discussion

The panoramic metabolite profile and nutritional composition of multiplier onion enhance our understanding and utilization of its nutritional and medicinal values. Here, widely targeted metabolomics analysis identified 659 metabolites in multiplier onion bulbs, with flavonoids and essential amino acids demonstrating particularly high abundance. In contrast, previous studies on common onions that utilized LC/ESI-QTOF-MS, GC-MS and UPLC-MS methods identified only over 100 metabolites, which was significantly fewer than the number of metabolites identified in this study. This finding demonstrated that the widely targeted metabolomics was a highly efficient approach for analyzing multiplier onions.

Flavonoids in plants serve various biological functions, including defense against abiotic and biotic stresses, regulation of male fertility, mediation of signaling pathways, and facilitation of auxin transport [26,27]. Moreover, flavonoids possess significant health-promoting properties in humans. Previous studies have established that common onion contains substantial quantities of flavonoids effective against cardiovascular disease, diabetes, bacterial infections, hyperlipidemia, and cancer [28]. Similarly, our study found that multiplier onion bulbs were rich in flavonoids, particularly quercetin, kaempferol, naringenin, isorhamnetin, and numerous derivatives such as quercetin-3-*O*-xyloside, quercetin-3-*O*-arabinoside, quercetin-4-*O*-glucoside, quercetin-3-*O*-galactoside, and kaempferol-3-*O*-glucoside. These compounds demonstrate potent biological activities, indicating the therapeutic potential of multiplier onion in anti-inflammatory and immune modulation, antioxidant and anti-aging processes, cardiovascular protection, and anti-tumor mechanisms. For example, the high content of quercetin exhibits potent anti-hypercholesterolemic properties, suggesting its potential as an effective anti-hypercholesterolemic agent [29].

Multiplier onion bulbs were found to contain abundant amino acids and their derivatives. Among these, eight of the nine essential amino acids—valine, threonine, leucine, isoleucine, lysine, methionine, histidine, and tryptophan—were detected in the bulbs of multiplier onion. Essential amino acids cannot be synthesized de novo by humans or animals and must be obtained through dietary sources. Although crucial for nutrition, several essential amino acids are often present in limiting quantities in major global crops [30]. The presence of these eight amino acids indicated that multiplier onion bulbs served as a significant source of essential amino acids. Furthermore, 15 vitamins were identified, primarily comprising B vitamins such as vitamin B2, B3, B5, B7, and B9, in addition to vitamin E and vitamin K2. These findings suggested that multiplier onions were rich in nutrients and functional ingredients that promote human health and aid in disease treatment.

Although some studies have reported the nutrient composition of multiplier onion, little information is available about the comparison of the metabolite composition differences among multiplier onion accessions. Among the five core multiplier onion accessions, the metabolite profiles exhibited distinct differences. For instance, the Aca-233 accession demonstrated a high accumulation of most amino acids and their derivatives, while both the Aca-148 and Aca-233 accessions displayed elevated levels of flavonoids. The DAMs were significantly enriched in amino acid biosynthesis and metabolism, and phenylpropanoid biosynthesis, as well as flavone and flavonol biosynthesis. Correspondingly, the DEGs were significantly enriched in flavonoid biosynthesis and amino acid biosynthesis and metabolism, aligning with the metabolomic analysis. These results indicated substantial variations in flavonoids, amino acids, and their derivatives across different multiplier onion accessions. Therefore, it is essential to screen for varieties with high nutritional value from the abundant germplasm resources available.

Modern molecular breeding technology represents an effective approach to significantly enhance breeding efficiency for asexually propagated crops [31,32], necessitating the identification of key genes that control target traits. In this study, several vital candidate genes were screened through a joint analysis of DAMs and DEGs. Notably, several correlated genes previously reported to be involved in flavonoid synthesis were identified, including *flavonoid 3′-hydroxylase* (*Aca_transcript_11093*) [33], *flavanone 3-hydroxylase* (*Aca_transcript_81295*, *Aca_transcript_17236*) [34], *flavonol synthase* (*Aca_transcript_17453*) [35], *glutathione S-transferase* (*Aca_transcript_21608*) [36], and *cytochrome P450* (*Aca_transcript_71174*, *Aca_transcript_56523*) [37,38]. Additionally, some correlated genes associated with amino acid synthesis, transport, and metabolism were identified, including *amino acid transporter 1-like* (*Aca_transcript_57289*) [39], *amino acid permease 3* (*AAP3*, *Aca_transcript_12428*), *AAP7* (*Aca_transcript_9530*) [40], and *isoaspartyl peptidase/L-asparaginase 1-like* (*Aca_transcript_29368*) [41]. These findings provided important candidate genes for quality improvement through molecular breeding.

## 5. Conclusions

This study integrated widely targeted metabolomics and RNA-seq transcriptomics to comprehensively examine metabolic and transcriptomic profiling. Metabolic profiling identified 659 metabolites in the bulbs of multiplier onion, with particularly high abundance of flavonoids and essential amino acids. The DAMs and DEGs were significantly enriched in pathways related to amino acid biosynthesis and metabolism, as well as flavonoid biosynthesis. The joint analysis of DAMs and DEGs elucidated the relationships between metabolites and gene expression, identifying candidate genes responsible for regulating the content of lipids, flavonoids, phenolic acids, amino acids and their derivatives. These findings provide insights into the nutrient metabolism components of multiplier onion and offer a foundation for the selection and improvement of high-quality multiplier onion varieties.

## Data Availability

The raw Illumina RNA sequencing data have been deposited into the BIG Submission Genome Sequence Archive (GSA) under the project accession number CRA028808. The other original contributions presented in this study are included in this article/Supplementary Materials.

## Supplementary Materials

The following supporting information can be downloaded at: https://www.mdpi.com/article/10.3390/foods14193290/s1, Table S1. A total of 659 metabolites identified in the 5 accessions of multiplier onion; Table S2. Differentially accumulated metabolites across five accessions of multiplier onion; Table S3. Information of significant correlation between differentially accumulated metabolites and differentially expressed genes. Figure S1. Total Ion Chromatogram (TIC) of all samples; Figure S2. MS/MS spectra of four metabolites.
