# Supplementary material for: Comprehensive Characterization of Metabolites in Multiplier Onion Bulbs and Identification of Regulatory Genes for Nutritional Improvement
Source: Foods. 2025 Sep 23;14(19):3290. doi: 10.3390/foods14193290 (PMC12523680; doi:10.3390/foods14193290)
Supplement: Supplementary file 1 [file foods-14-03290-s001.zip › Supplementary Figures.pptx]

## Slide 1
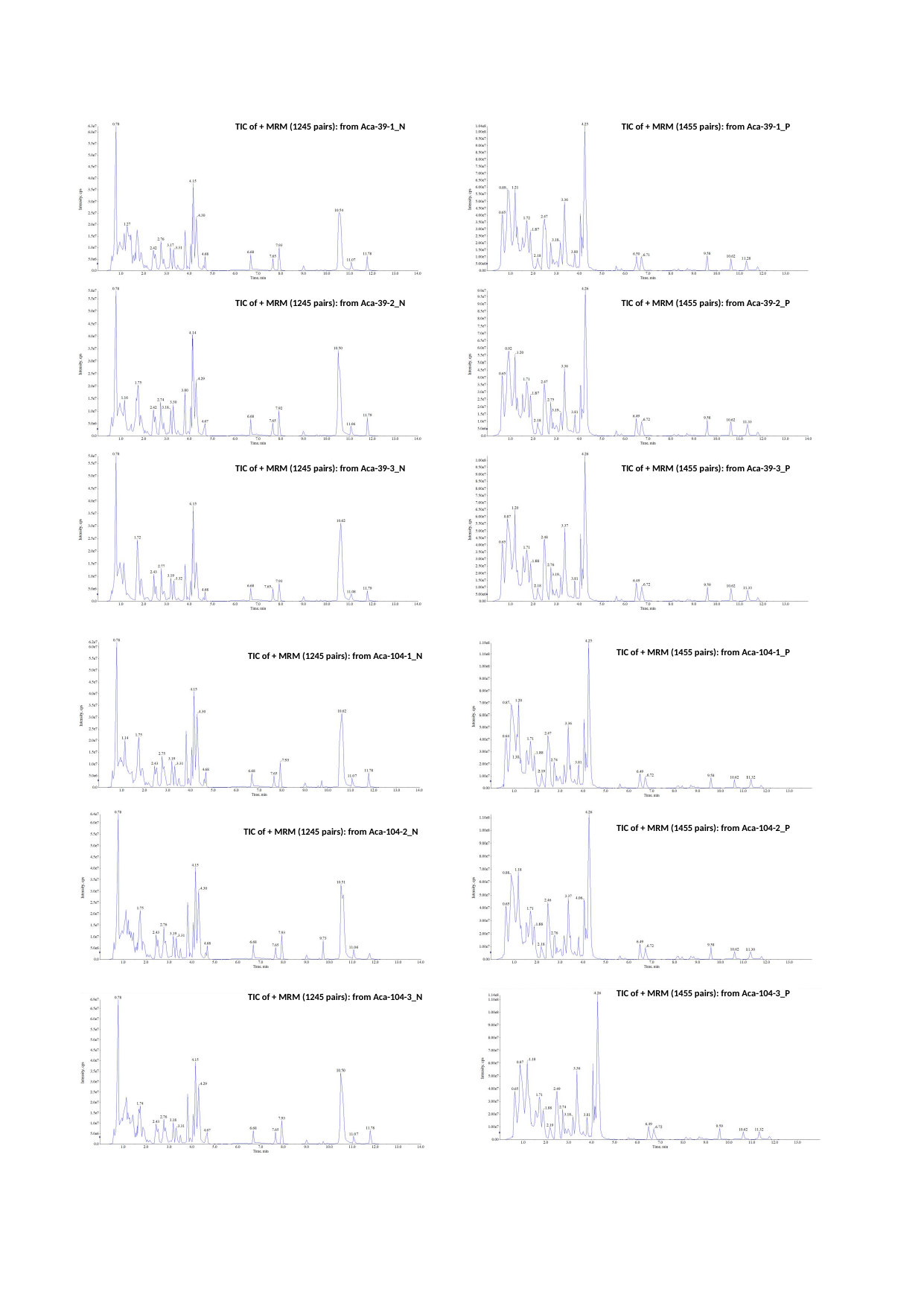

TIC of + MRM (1245 pairs): from Aca-39-1_N
TIC of + MRM (1455 pairs): from Aca-39-1_P
TIC of + MRM (1245 pairs): from Aca-39-2_N
TIC of + MRM (1455 pairs): from Aca-39-2_P
TIC of + MRM (1245 pairs): from Aca-39-3_N
TIC of + MRM (1455 pairs): from Aca-39-3_P
TIC of + MRM (1455 pairs): from Aca-104-1_P
TIC of + MRM (1245 pairs): from Aca-104-1_N
TIC of + MRM (1455 pairs): from Aca-104-2_P
TIC of + MRM (1245 pairs): from Aca-104-2_N
TIC of + MRM (1455 pairs): from Aca-104-3_P
TIC of + MRM (1245 pairs): from Aca-104-3_N

## Slide 2
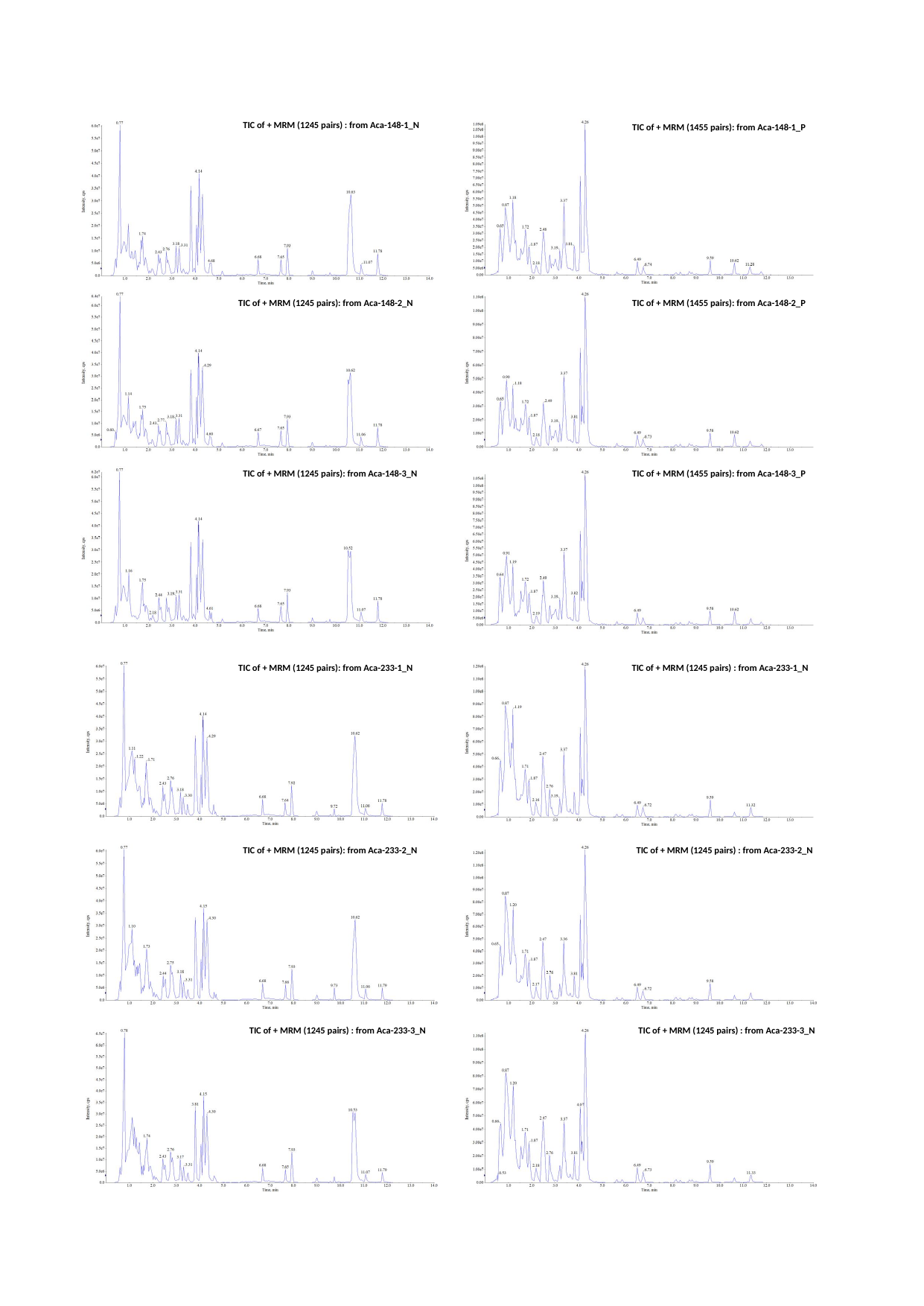

TIC of + MRM (1245 pairs) : from Aca-148-1_N
TIC of + MRM (1455 pairs): from Aca-148-1_P
TIC of + MRM (1245 pairs): from Aca-148-2_N
TIC of + MRM (1455 pairs): from Aca-148-2_P
TIC of + MRM (1455 pairs): from Aca-148-3_P
TIC of + MRM (1245 pairs): from Aca-148-3_N
TIC of + MRM (1245 pairs): from Aca-233-1_N
TIC of + MRM (1245 pairs) : from Aca-233-1_N
TIC of + MRM (1245 pairs): from Aca-233-2_N
TIC of + MRM (1245 pairs) : from Aca-233-2_N
TIC of + MRM (1245 pairs) : from Aca-233-3_N
TIC of + MRM (1245 pairs) : from Aca-233-3_N

## Slide 3
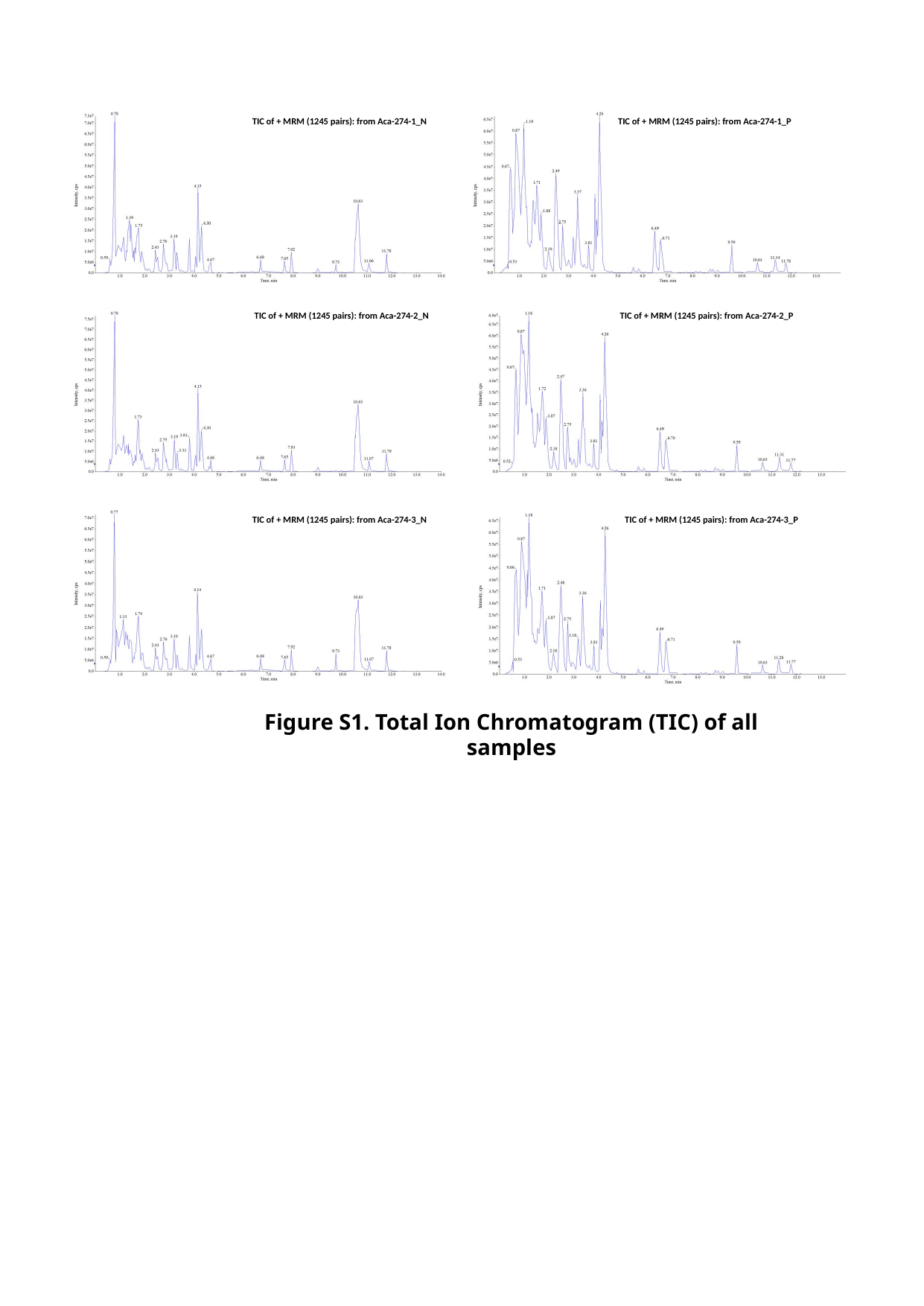

TIC of + MRM (1245 pairs): from Aca-274-1_N
TIC of + MRM (1245 pairs): from Aca-274-1_P
TIC of + MRM (1245 pairs): from Aca-274-2_N
TIC of + MRM (1245 pairs): from Aca-274-2_P
TIC of + MRM (1245 pairs): from Aca-274-3_N
TIC of + MRM (1245 pairs): from Aca-274-3_P
Figure S1. Total Ion Chromatogram (TIC) of all samples

## Slide 4
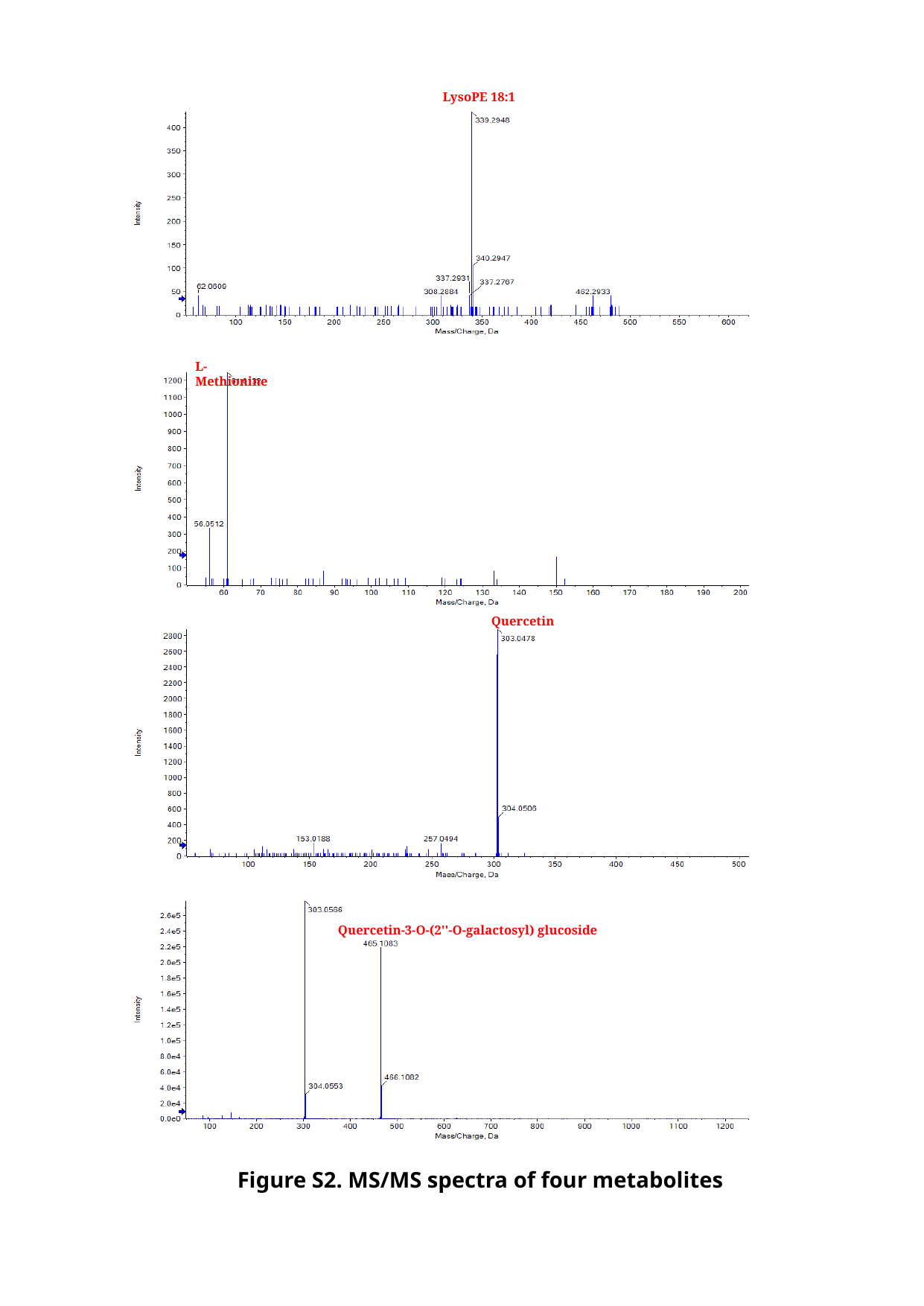

LysoPE 18:1
L-Methionine
Quercetin
Quercetin-3-O-(2''-O-galactosyl) glucoside
Figure S2. MS/MS spectra of four metabolites
